# Dissecting the Effects of Selection and Mutation on Genetic Diversity in Three Wood White (*Leptidea*) Butterfly Species

**DOI:** 10.1093/gbe/evz212

**Published:** 2019-10-03

**Authors:** Venkat Talla, Lucile Soler, Takeshi Kawakami, Vlad Dincă, Roger Vila, Magne Friberg, Christer Wiklund, Niclas Backström

**Affiliations:** 1 Department of Evolutionary Biology, Evolutionary Biology Centre (EBC), Uppsala University, Sweden; 2 Department of Medical Biochemistry and Microbiology, National Bioinformatics Infrastructure Sweden (NBIS), Science for Life Laboratory, Uppsala, Sweden; 3 Department of Ecology and Genetics, University of Oulu, Finland; 4 Institut de Biologia Evolutiva (CSIC-UPF), Barcelona, Spain; 5 Department of Biology, Biodiversity Unit, Lund University, Sweden; 6 Department of Zoology, Division of Ecology, Stockholm University, Sweden

**Keywords:** adaptation, speciation, *Leptidea*, Lepidoptera, cryptic species, selection

## Abstract

The relative role of natural selection and genetic drift in evolution is a major topic of debate in evolutionary biology. Most knowledge spring from a small group of organisms and originate from before it was possible to generate genome-wide data on genetic variation. Hence, it is necessary to extend to a larger number of taxonomic groups, descriptive and hypothesis-based research aiming at understanding the proximate and ultimate mechanisms underlying both levels of genetic polymorphism and the efficiency of natural selection. In this study, we used data from 60 whole-genome resequenced individuals of three cryptic butterfly species (*Leptidea* sp.), together with novel gene annotation information and population recombination data. We characterized the overall prevalence of natural selection and investigated the effects of mutation and linked selection on regional variation in nucleotide diversity. Our analyses showed that genome-wide diversity and rate of adaptive substitutions were comparatively low, whereas nonsynonymous to synonymous polymorphism and substitution levels were comparatively high in *Leptidea*, suggesting small long-term effective population sizes. Still, negative selection on linked sites (background selection) has resulted in reduced nucleotide diversity in regions with relatively high gene density and low recombination rate. We also found a significant effect of mutation rate variation on levels of polymorphism. Finally, there were considerable population differences in levels of genetic diversity and pervasiveness of selection against slightly deleterious alleles, in line with expectations from differences in estimated effective population sizes.

## Introduction

Theory predicts that both the level of genetic diversity (Kimura and Crow 1964; Kimura 1983) and the rate of adaptive change (Gillespie 2001; Eyre-Walker and Keightley 2007; Leffler et al. 2012; Lanfear et al. 2014; Galtier 2016) can vary across populations and species as a consequence of differences in effective population size (*N*_e_). The level of neutral genetic diversity in a population is determined by the mutation rate (*μ*) and the loss of genetic variants due to genetic drift (Kimura and Crow 1964; Kimura 1983). Both the total number of novel mutations entering a population and the effect of genetic drift are dependent on *N*_e_, resulting in an equilibrium genetic diversity level which is a product of *μ* and *N*_e_ (4 × *N*_e_ × *μ* in diploids), which translates to a higher expected level of genetic diversity in larger populations (Charlesworth 2009). Empirical studies using protein and/or DNA-sequence population data have found a positive correlation between the level of diversity and estimated population size, but that the diversity range interval was much narrower than expected given the inferred variation in population size across species (Lewontin 1974; Ohta and Gillespie 1996; Leffler et al. 2012). Moreover, the fraction of amino acid substitutions driven to fixation by positive selection is predicted to be highest in populations and species with large *N*_e_. Analyses comparing evolutionary rates in species with large (*Drosophila*) and small *N*_e_ (humans) indeed indicated that the proportion of adaptive substitutions driven to fixation by positive selection has been substantially larger in flies than in humans (Eyre-Walker 2006; Eyre-Walker and Keightley 2009). These observations were corroborated by subsequent efforts in organisms with both comparatively small (Boyko et al. 2008; Gossmann et al. 2010; Loire et al. 2013) and large to very large *N*_e_ (Charlesworth and Eyre-Walker 2006; Halligan et al. 2010; Carneiro et al. 2012; Tsagkogeorga et al. 2012) and in meta-analyses involving sets of taxa with variation in *N*_e_ (Gossmann et al. 2012; Phifer-Rixey et al. 2012; Rousselle et al. 2019). However, yeast and maize, which presumably have very large *N*_e_ (>10^7^ and >10^5^, respectively), show limited evidence for adaptive processes driving gene evolution (Tsai et al. 2008; Liti et al. 2009; Strasburg et al. 2011; Gossmann et al. 2012). Hence, not all empirical data support that *N*_e_ is the main determinant of the level of genetic diversity or the rate of adaptive evolution, and basic population genetic models cannot explain why both the rate of adaptive evolution and the maintenance of neutral genetic diversity are lower than expected in large populations (Jensen and Bachtrog 2011; Galtier 2016).

One solution to this discrepancy may be that selection on linked sites via genetic hitch-hiking (Maynard Smith and Haigh 1974) and/or background selection (Charlesworth et al. 1993) plays a larger role than previously accounted for (Hill and Robertson 1966; Leffler et al. 2012; Martin et al. 2016). The effects of such “linked selection” is dependent on joint effects of *μ*, *N*_e_, the density of targets of natural selection, the relative frequency and fitness effects of adaptive and deleterious mutations and the recombination rate (Mugal et al. 2013; Romiguier et al. 2014). Quantification of rates and patterns of adaptive microevolutionary change is crucial to understand the generation and maintenance of biodiversity and for predicting the evolutionary potential of extant species and populations (Lynch and Lande 1998; Gillespie 2001; Leffler et al. 2012; Lanfear et al. 2014). For this, extensive additional analyses of rates of adaptation and levels of genetic diversity are needed across a large number of taxonomic groups in general (Leffler et al. 2012), and for specific lineages of conservation concern in particular (Lynch and Lande 1998).

The three cryptic butterfly species, wood white (*Leptidea sinapis*), Real’s wood white (*L. reali*), and cryptic wood white (*L. juvernica*), have distribution ranges covering a major part of western and central Eurasia (Dincă et al. 2011). *Leptidea juvernica* diverged from *L. reali* and *L. sinapis* 2.5–3.5 Ma and the latter two species diverged 1–2 Ma. The three species are virtually identical in external morphology but can be distinguished via joint analyses of genital- and karyotype structure and/or molecular analysis (Dincă et al. 2011; Talla et al. 2017, 2019). Reproductive isolation between species is complete or near complete, potentially both via female mate choice (Friberg, Vongvanich, et al. 2008; Dincă et al. 2013) and karyotypic incompatibilities (Lukhtanov et al. 2018; Talla et al. 2019). Species-specific adaptations related to diapause propensity, phenology, utilization of host plants and mating behavior have been observed, although there is no diagnostic pattern since distinctive ecotypes with complex differences in life-history strategies and habitat preferences also occur within *L. sinapis* and *L. juvernica* (Friberg, Olofsson, et al. 2008; Friberg and Wiklund 2009, 2010; Friberg et al. 2013). In addition, *L. sinapis* populations differ in chromosome numbers in an exceptional cline across western Eurasia where the karyotype setup varies from 2*n* = 57, 58 in the north (Sweden) and the east (Kazakhstan) to 2*n* = 106–108 in southwest (Spain) (Dincă et al. 2011; Lukhtanov et al. 2011; Šíchová et al. 2015). The karyotype extremes in *L. sinapis* are partly reproductively isolated, showing evidence of considerable hybrid breakdown when crossed (Lukhtanov et al. 2018). Recent genomic analyses suggest that the wood whites have differentiated without any postdivergence gene flow (Talla et al. 2019). This study also suggested that lineage specific, weak selection, and random genetic drift have been the main drivers of species divergence. All three species have lower genetic diversity (∼0.2–0.3%) and a larger number of transposable elements than most other butterflies, indicating comparatively low long-term *N*_e_ (Talla et al. 2017, 2019).

In this study, we combined previously available genomic resources (genome assembly, whole genome resequencing data of 60 individuals, transcriptome data from multiple developmental stages and both sexes) to generate novel coding sequence annotation information and recombination rate data in order to examine the prevalence of natural selection in species and geographically distinct populations with and without karyotype differences. Our main aims were to estimate the effects of mutation, selection on linked sites (recombination and density of genes) and base composition on regional variation in genetic diversity, and quantify the adaptive potential in *Leptidea* in general and between populations with apparent differences in ecology, behavior, and population size in particular.

## Materials and Methods

### Sampling, Sequencing, and Genotyping

Publicly available, whole genome resequencing data from 60 male individuals and RNA-seq data from multiple developmental stages (larva, pupa, imago) were used in this study. Detailed information about sampling, genome assembly, DNA- and RNA resequencing, and individual genotyping is available in Talla et al. (2017, 2019) and Leal et al. (2018). In brief, the sample set used for analysis included ten samples from each of six populations, representing three species of the *Leptidea* cryptic complex: *L. sinapis*, *L. reali*, and *L. juvernica*. *Leptidea sinapis* samples were collected in Sweden (LsSwe, 2*n* = 57, 58), Kazakhstan (LsKaz, 2*n* = 56–58), and Spain (LsSpa, 2*n* = 106–108), hence covering both the extreme karyotypes of the species and populations located geographically far apart but with similar karyotypes. *Leptidea reali* was collected in Spain (LrSpa), and *L. juvernica* samples were collected in Ireland (LjIre) and Kazakhstan (LjKaz). Genomic DNA libraries from all samples were individually barcoded and sequenced using the Illumina Hi-seq technology to obtain paired-end reads with an average coverage of ∼12× for each sample. A reference genome for an inbred male Swedish *L. sinapis* was assembled with both mate-pair and paired-end reads (Talla et al. 2017). DNA sequencing reads from each sample were trimmed for adapters and low-quality bases and mapped to the *L. sinapis* reference genome (Talla et al. 2019). Polymorphisms were identified using a combination of variant callers to generate a “golden set” of SNPs (Li et al. 2009; McKenna et al. 2010; Garrison and Marth 2012), which was used as input for final variant calling in GATK (McKenna et al. 2010; Talla et al. 2019). For the analysis performed in this study, only SNPs that were covered at least two times in all individuals in each respective population/species were used and population- and species-specific allele frequencies were estimated for these SNPs using VCFtools (Danecek et al. 2011). Transcriptome data were generated for 36 Swedish *L. sinapis* individuals representing three developmental stages and both sexes (Leal et al. 2018) and 12 representative RNA-seq libraries from this data set were used for de novo annotation of the *L. sinapis* genome assembly (see below).

### Gene Annotation

The *L. sinapis* genome assembly (Talla et al. 2017) was annotated based on a standardized pipeline developed by the NBIS team at the SciLife Laboratory in Uppsala. Briefly, the annotation included the following steps; collection of reference proteins from databases, assembly of RNA-seq data from 12 *L. sinapis* transcriptome libraries, representing larvae, pupae, and adults of both males and females (see Leal et al. 2018 for details), annotation inference using both direct transcriptome data and ab initio predictions, functional annotation and creation of a WebApollo portal to allow for manual curation (for a detailed description of the annotation process, see supplementary methods, Supplementary Material online).

### Recombination Rate, Gene Density, and GC-Content Estimates

To estimate the population recombination rate (ρ  = 4 × *N*_e_ × *r*, where *r* is the recombination rate per base-pair per generation), we first reconstructed haplotypes of SNPs for each population separately by ShapeIt (Delaneau et al. 2012). We used only biallelic SNPs for which >90% of individuals were genotyped. Then ρ was estimated for each of the six populations using LDhelmet (Chan et al. 2012). LD helmet uses patterns of linkage disequilibrium (LD) between SNP pairs to estimate the population scaled recombination rate (rho). The analysis operates on information about ancestral allele frequencies in combination with a substitution model to generate a recombination map along a chromosome. Since there was no suitable outgroup species available for estimating ancestral allele frequencies, we used the stationary distribution of the mutation matrix. Sites were excluded if the variant quality was <15 and the mapping quality <20. This filtering reduced the number of SNPs only marginally (<0.01% of SNPs filtered out in all populations, supplementary table 1, Supplementary Material online). A minimum coverage threshold of 3× per site in at least five individuals in each respective population was applied. Prior probabilities for the ancestral allele and the three alternative alleles were set at 0.97 and 0.01, respectively. We used a nucleotide substitution matrix in *Drosophila melanogaster* (Chan et al. 2012) and ran five independent rjMCMC simulations for each population. Each simulation was run for 2,000,000 iterations with a burn-in of an additional 200,000 iterations. Population recombination (ρ) estimates were averaged across the five runs. Based on results from a recent simulation analysis (Kawakami et al. 2017), a block penalty of 10 was applied to minimize overfitting. Note that we were not interested in identifying recombination hotspots (narrow genomic regions with extremely high recombination rate) but were interested in broad-scale variation of recombination rate, which is not particularly sensitive to the choice of the parameters. To quantify the variation in ρ across the genome, weighted averages were calculated in 100 kilobase (kb) nonoverlapping windows across scaffolds. The density of targets of natural selection (number of coding sequence nucleotides/total number of nucleotides with sequence information in the window, including repeats) was estimated for the corresponding 100 kb windows (note that this ignores potential functional noncoding sequences that might be under selective pressure). The proportion of guanine and cytosine bases (GC-content) in each window was estimated with an in-house developed python script (https://github.com/venta380/Leptidea_selection_project).

A multiple linear regression as implemented in the R (https://cran.r-project.org/) package *stats* was applied to investigate effects of mutation, recombination, gene density, and base composition on regional variation in genetic diversity. The rationale behind choosing these parameters is that linked selection should be affected both by the number of targets of selection (gene density) and the recombination rate (Mugal et al. 2015). To account for variation in base composition—a consequence of significantly higher GC-content in coding—than in noncoding sequence (see results)—GC-content was also included as an explanatory variable. Parameter estimates were calculated in 100 kb windows in each population separately to assess potential variation in effects on linked selection. The parameter settings included separate analyses with independent *d*_S_, ρ, gene density, and GC-content + interaction effects between different explanatory variables.

### Diversity Estimates and Assessment of Natural Selection

Average pairwise nucleotide diversity (θ_π_), diversity in 4-fold degenerate (4D) sites (π_4D_), ratios of nonsynonymous polymorphisms per nonsynonymous site to synonymous polymorphisms per synonymous site (*p*_N_/*p*_S_) and nonsynonymous substitutions per nonsynonymous site to synonymous substitutions per synonymous site (*d*_N_/*d*_S_ or ω) were calculated across the genome in windows of 100 kb for all three species using in-house developed scripts (see Data Deposition). Relative nucleotide diversity (θ_π_^Z^) for each window was calculated by standardizing the window θ_π_ to the mean genome wide θ_π_ of the species (Equation 1).

(1) $$\theta_{\pi }^{Z}=\;\frac{{{\mathrm{Window}\;\theta }_{\pi }-\;\mathrm{Genome}\;\mathrm{wide}\;\theta }_{\pi }}{{\mathrm{SD}\;\mathrm{of}\;\mathrm{genome}\;\mathrm{wide}\;\theta }_{\pi }}$$

If a window had θ_π_^Z^ >0 it was classified as a “high-diversity region” and if θ_π_^Z^ was <0 it was classified as a “low-diversity region.” We only included windows that contained ≥1,000 coding positions and a coverage of ≥ 50% of the sites in all individuals in a population. This filtering resulted in 4,210 windows (421 Mb) being retained, containing 16.5 Mb of coding sequence. The proportion of adaptive nonsynonymous substitutions (α) was calculated using DFE-alpha version 2.16, a method incorporating segregation of slightly deleterious nonsynonymous polymorphisms (Keightley and Eyre-Walker 2007). In this analysis, 2nd codon positions were defined as selected sites and 4D sites as neutral and variance estimates were generated by bootstrapping (200 iterations).

## Results

### Annotation

In total, 15,598 complete gene sequences were obtained from the annotation process of the *L. sinapis* genome assembly. Of these, 8,816 genes were found to be 1:1 orthologs to genes with available functional annotation from Uniprot and Swiss-Prot, whereas the corresponding number for Flybase was 8,826 genes. The average CDS length was 1,134 base pairs (bp) and 72.1% of the genes had predicted 5′- and 3′-untranslated regions. The protein coding genes covered 3.7% of the genome assembly (supplementary table 2, Supplementary Material online). The classification of functional categories using a set of different databases resulted in functional information for 10,857 of the 15,598 annotated genes; 4,741 of predicted *L. sinapis* genes hence lack functional information (supplementary table 3, Supplementary Material online).

### Variation in Diversity between Site Categories Linked to Base Composition

**Fig. 1.**
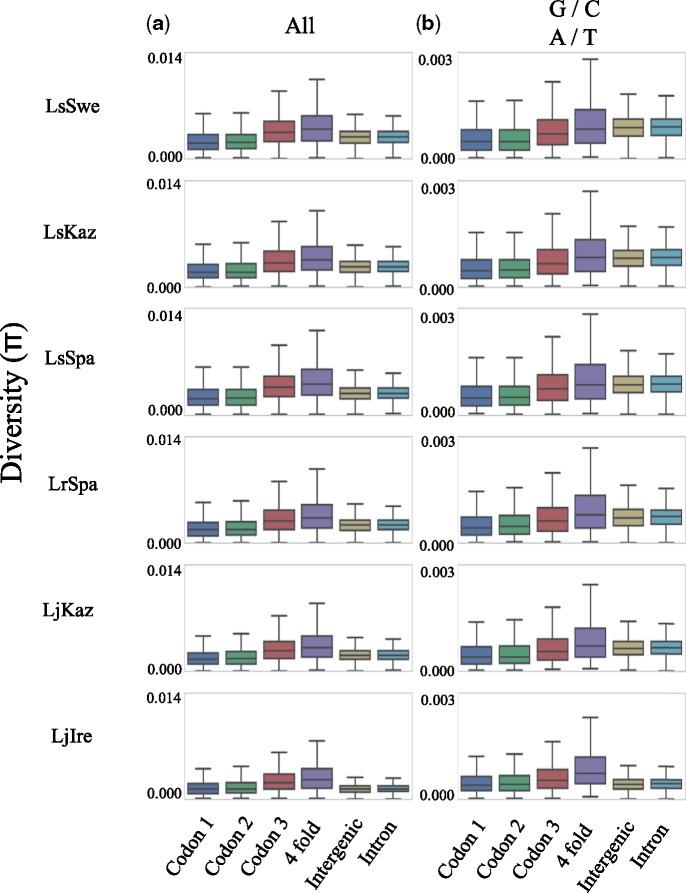
—Boxplots showing the nucleotide diversity at different site categories calculated using all (vertical panel *a*), or only weak to weak (A/T) and strong to strong (G/C) polymorphisms (vertical panel *b*).

**Fig. 2.**
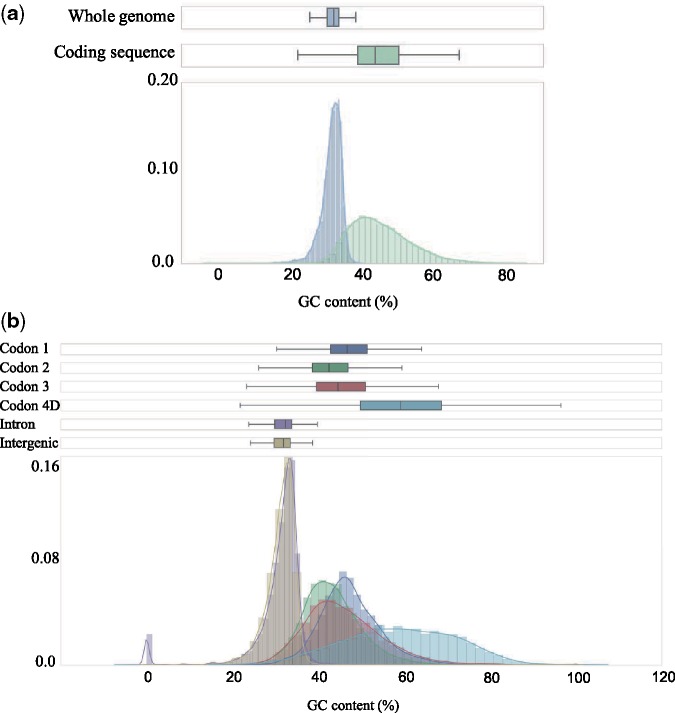
—The distribution of GC-content (%) across 100 kb windows for protein coding- and noncoding sequences (*a*) and for separate site categories (*b*).

We assessed how θ_π_ varied between different site categories. Based on the annotation information, sites were classified into the following categories: 1st, 2nd, or 3rd codon position, 4D sites (note that this is a subset of the 3rd codon position), intronic or intergenic. This was done for all six populations individually to accommodate for differences in demographic history across populations within species. In line with general expectations from an effect of purifying selection, the observed θ_π_ was lower in 1st and 2nd than in 3rd codon positions, but higher in 4D sites than in intergenic and intronic sites (fig. 1). Given that base composition may affect the mutation rate and the level of polymorphism (Gojobori et al. 1982; Duchêne et al. 2015), the GC-content of each category was estimated. We found that the GC-content varied extensively among positions, being considerably higher in coding regions (44.9 ± 8.4%) than in introns and intergenic sequences (31.6 ± 2.7%) and the variation was noticeably different across site categories, with the largest difference between 4D sites (58.9 ± 13.1%) and introns (30.7 ± 6.5%) (fig. 2 and supplementary table 4, Supplementary Material online). To take the biased base composition across sites into account, θ_π_ for different site categories was estimated using only weak to weak (A/T) and strong to strong mutations (G/C), which should not affect the GC-content. For these polymorphisms, θ_π_ was again lowest in the 1st and 2nd codon positions, followed by 3rd codon positions. However, the diversity in introns and intergenic sequences was now similar to, or higher than the level in 4D sites. This was true for all populations, except for LjIre, which is the population with the lowest overall θ_π_ (fig. 1).

### Regional Variation in Genetic Diversity and Associations with Recombination, Selection, Gene Density, and Base Composition

The global population recombination rate (ρ) estimates were similar across all populations (mean ρ range across populations = 0.037–0.053) except in LjIre which had lower overall ρ (mean ρ = 0.016 ± 0.028, supplementary fig. 1, Supplementary Material online). Regional estimates for population pairs were only marginally positively correlated, with the exception of the populations LsSwe and LsKaz, where we found a significant positive correlation (Pearson’s *r* = 0.21, *P* value <0.001; supplementary fig. 2, Supplementary Material online). Following the expectation based on the role of linked selection, there was a weak but significant positive correlation between ρ and θ_π_ in all populations where LsSwe and LsKaz showed the strongest correlation, followed by LjKaz and LsSpa, whereas LjIre and LrSpa did not show such a correlation (supplementary fig. 3, Supplementary Material online).

**Fig. 3.**
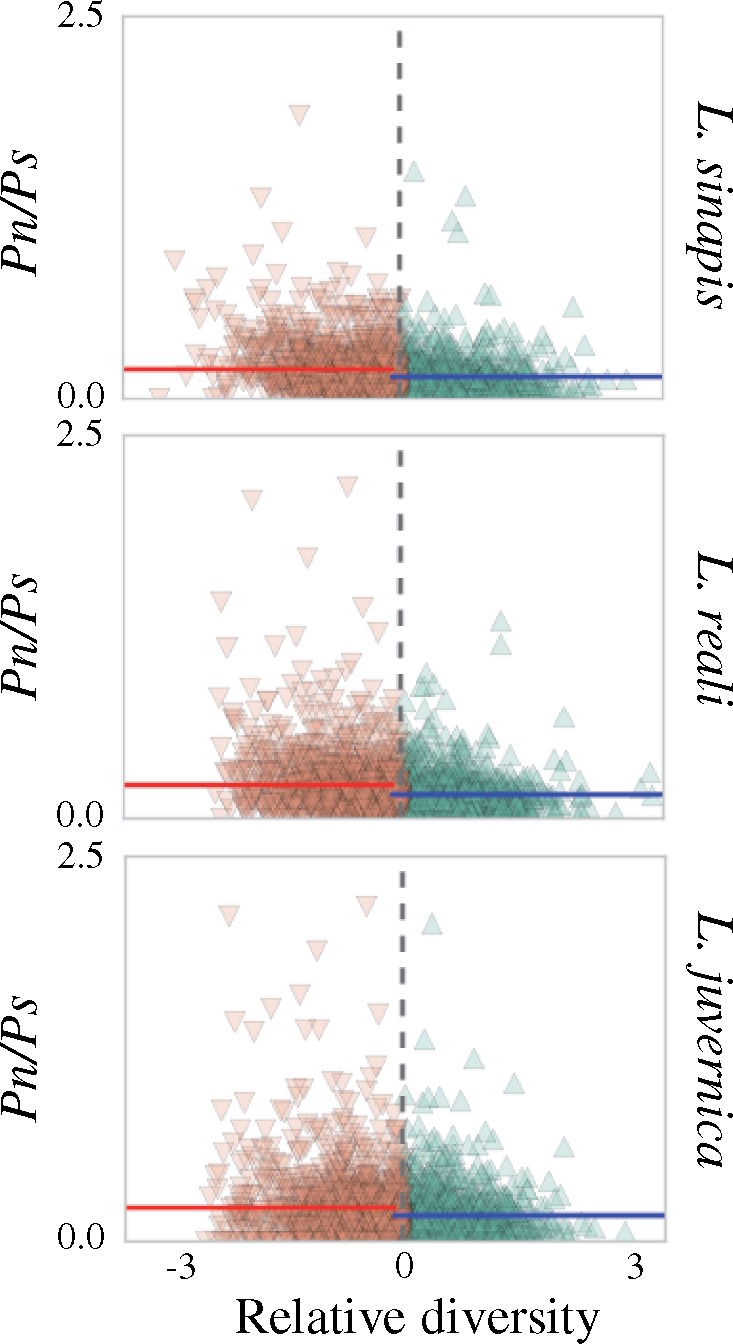
—Scatter plots showing the differences in average *p*_N_/*p*_S_ ratios in low- (brown) and high- (green) diversity regions for each species. Vertical colored lines show the mean *p*_N_/*p*_S_ of low- (red) and high- (blue) diversity regions, respectively. Note that diversity is calculated as the window-based estimate relative to the genomic average (*x* axis) and data points are therefore centered at 0.

In line with previous observations (Talla et al. 2019), the genome wide θ_π_ averaged across 100 kb windows was highest in *L. sinapis* (0.0031 ± 0.00087), intermediate in *L. reali* (0.0024 ± 0.00088) and lowest in *L. juvernica* (0.0016 ± 0.00049). To assess if regional variation in diversity was associated with density of targets of selection (gene density), recombination rate and/or base composition, θ_π_ was compared with the *p*_N_/*p*_S_ ratio, ω, ρ, and GC-content in 100 kb windows across the genome. For each species, genomic regions were divided into two categories, “high” and “low-diversity regions,” based on θ_π_^Z^ to quantify the effects of selection and base composition on diversity levels. In agreement with less efficient selection in low recombination regions, the low-diversity regions had significantly higher *p*_N_/*p*_S_ ratios (Mann–Whitney *U* tests, *P* values = *L. sinapis*: 1.8×10^−20^; *L. reali*: 5.8×10^−17^; *L. juvernica*: 4.8×10^−4^) than the high-diversity regions in all species (fig. 3; tables 1 and 2; and supplementary fig. 4, Supplementary Material online). Similar to the observation for polymorphism ratios, we observed a significantly higher ω in the low-diversity regions compared with the high-diversity regions in all species (tables 1 and 2; supplementary fig. 5, Supplementary Material online).

**Table 1:** Ratios of Nonsynonymous to Synonymous Polymorphisms (*p*_N_/*p*_S_) in Low- (low) and High-Diversity (high) Regions across the Genome in All Species

Species	Low	High	*P* value
*L. sinapis*	0.22±0.29	0.16±0.21	1.8×10^−20^
*L. reali*	0.24±0.20	0.18±0.13	5.8×10^−19^
*L. juvernica*	0.22±0.28	0.18±0.20	4.8×10^−4^

Note.—*P* values for the Mann–Whitney *U* test are given for each respective comparison.

**Table 2:** Ratios of Nonsynonymous to Synonymous Substitutions (*d*_N_/*d*_S_ or ω) in Low- (low) and High-Diversity (high) Regions across the Genome in All Species

Species	Low	High	*P* value
*L. sinapis*	0.15±0.26	0.11±0.25	3.9×10^−3^
*L. reali*	0.21±0.31	0.18±0.28	4.2×10^−4^
*L. juvernica*	0.19±0.36	0.13±0.28	1.8×10^−4^

Note.—*P* values for the Mann–Whitney *U* test are given for each respective comparison.

**Fig. 4.**
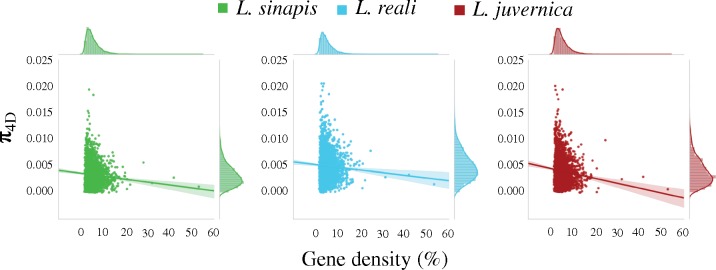
—The relationship between nucleotide diversity at 4-fold degenerate sites (π_4D_) and gene density (proportion of protein coding/exonic sites in a window in %) calculated across 100 kb windows in all three species.

We assessed if the observed differences in *p*_N_/*p*_S_ ratios and ω could be a consequence of differences in the density of targets of natural selection. When low- and high-diversity regions were compared, we found that the proportion of exon sequences (gene density) was significantly higher in low-diversity regions (supplementary fig. 6 and table 5, Supplementary Material online). In a similar analysis, investigating the relationship between π_4D_ and gene density, we found a significant negative correlation in all species (*L. sinapis*: Pearson’s *r* = −0.058, *P* value = 0.004; *L. reali*: Pearson’s *r* = −0.11, *P* value <0.001, *L. juvernica*: Pearson’s *r* = −0.083, *P* value <0.001; fig. 4). The GC-content was higher in high-, than in low-diversity regions (supplementary fig. 7 and table 6, Supplementary Material online).

To characterize effects of mutation and linked selection on regional genetic diversity, we applied a multilinear regression (MLR) analysis using genetic diversity as response variable and recombination rate, base composition, gene density, and mutation rate (lineage-specific *d*_S_) as explanatory variables. The independent effects of each explanatory variable were supported by variance inflation factors close to 1 (supplementary table 7, Supplementary Material online). In line with the expected impact of linked selection and mutation rate variation on nucleotide diversity, the MLR showed that ρ, gene density, and *d*_S_ independently explained a significant part of the variance in π_4D_ in several populations, whereas interaction effects and base composition were mostly insignificant (table 3 and supplementary table 7, Supplementary Material online).

**Table 3:** Summary of the Multiple Linear Regression Analysis Where Base Composition (GC), Recombination Rate (ρ), Gene Density (GD), and Mutation Rate (*d*_S_) Were Used as Explanatory Variables for Variation in Genetic Diversity at 4-Fold Degenerate Coding Positions (π_4D_)

Population	Parameter	Estimate	SE	*t* Value	Pr(>|*t*|)
LsSwe	GC	−7.94×10^−5^	4.94×10^−4^	−0.16	0.872
	ρ	2.12×10^−3^	5.06×10^−4^	4.18	2.98×10^−5^***
	GD	−7.40×10^−4^	5.32×10^−4^	−1.39	0.165
	*d*_S_	−2.41×10^−2^	2.72×10^−2^	−0.89	0.374
LsKaz	GC	1.51×10^−4^	4.98×10^−4^	0.30	0.762
	ρ	9.59×10^−4^	5.09×10^−4^	1.88	0.060
	GD	−7.45×10^−4^	5.35×10^−4^	−1.39	0.164
	*d*_S_	−2.29×10^−2^	2.71×10^−2^	−0.84	0.399
LsSpa	GC	3.52×10^−4^	4.86×10^−4^	0.73	0.469
	ρ	5.22×10^−3^	5.01×10^−4^	10.42	<2.0×10^−16^***
	GD	2.78×10^−4^	5.22×10^−4^	0.53	0.594
	*d*_S_	−6.19×10^−2^	2.68×10^−2^	−2.32	2.1×10^−2^*
LrSpa	GC	−1.05×10^−4^	6.67×10^−4^	−0.16	0.875
	ρ	1.53×10^−3^	6.62×10^−4^	2.31	2.09×10^−2^*
	GD	−1.28×10^−3^	6.97×10^−4^	−1.84	6.62×10^−2^
	*d*_S_	−1.08×10^−1^	1.85×10^−2^	−5.81	7.17×10^−9^***
LjKaz	GC	−7.35×10^−4^	5.34×10^−4^	−1.38	0.169
	ρ	4.30×10^−3^	5.64×10^−4^	7.63	3.52×10^−14^***
	GD	−1.32×10^−3^	5.74×10^−4^	−2.30	2.15×10^−2^*
	*d*_S_	−8.69×10^−2^	2.12×10^−2^	−4.09	4.42×10^−5^***
LjIre	GC	−4.64×10^−4^	5.35×10^−4^	−0.868	0.385
	ρ	6.90×10^−4^	5.70×10^−4^	1.21	0.226
	GD	−1.14×10^−3^	5.69×10^−4^	−2.003	0.045*
	*d*_S_	−6.06×10^−2^	2.11×10^−2^	−2.875	0.004**

Note.—Variance inflation factors for explanatory variables and interaction effects are presented in supplementary table 7, Supplementary Material online. The significance level of the variables are represented by the symbol ‘*’. ‘***’ represents highly significant, ‘**’ represents moderately significant and ‘*’ represents slightly significant.

### Rate of Adaptation

The estimated proportion of adaptive nonsynonymous changes (α) in *L. sinapis* and *L. reali* (the parameter could not be estimated in *L. juvernica* since we are lacking a suitable outgroup species) was low as compared with taxa with larger *N*_e_. The proportion was slightly higher in *L. sinapis* (0.12 ± 0.04) than in *L. reali* (0.09 ± 0.04).

## Discussion

### General

We used whole genome sequences from 60 individuals of three different wood white species to examine the forces shaping variation in genetic diversity. The three *Leptidea* species showed a low level of genome-wide, neutral genetic variation (∼0.2–0.3%), considerably lower than the majority of other investigated butterfly taxa: for example, *Heliconius melpomene* (∼2%), *Papilio glaucus* (∼2.3%), *Pieris rapae* (∼1.5%), and *Phoebis sennae* (∼1.2%) (The Heliconius Genome Consortium 2012; Cong et al. 2016; Martin et al. 2016; Shen et al. 2016). This indicates reduced effective population sizes in all three *Leptidea* species compared with other butterflies. In line with the lower diversity level in *Leptidea*, we observed that the proportion of adaptive nonsynonymous substitutions (≈0.10) was lower than estimates from *Heliconius* butterflies (0.29–0.33; Martin et al. 2016). The rates in *Leptidea* are within the distribution of estimates from a large set of animal species but at the low end for invertebrates (Jensen and Bachtrog 2011; Messer and Petrov 2013; Galtier 2016). This supports that adaptive rates depend on *N*_e_, although this effect may be diluted over longer time scales due to a negative association between *N*_e_ and the proportion of beneficial mutations (Rousselle et al. 2019).

All three *Leptidea* species had reduced *p*_N_/*p*_S_ ratios and a slightly lower ω in high-diversity regions as compared with low-diversity regions. This supports an overall effect of purifying selection, reducing the allele frequency, and the probability of fixation of slightly deleterious mutations in high-diversity regions. It should be noted, however, that nonindependence between *p*_N_/*p*_S_ or ω on the one hand and θ_π_ on the other could inflate the significance level and the exact effects should therefore be treated with caution. Our analyses also show that regional variation in π_4D_ is determined by joint effects of mutation rate (*d*_S_), recombination rate, and gene density, but not by base composition. Hence, selection on linked sites acts to reduce genetic diversity and maintenance of genetic variation is dependent on the recombination rate and density of targets of selection, which corroborate recent findings, both in butterflies (Martin et al. 2016, 2019; Mackintosh et al. 2019) and other taxa (Cutter and Payseur 2013; Corbett-Detig et al. 2015; Castellano et al. 2019; Rettelbach et al. 2019). Hence, despite presumably low *N*_e_ in all three *Leptidea* species, selection on linked sites has been a considerable force underlying intragenomic variation in genetic diversity. Our results call for studies on the effects of natural selection on variation in genetic diversity across organisms should include populations that differ in recombination rate, population size, karyotype organization, and genome architecture (gene density), with background selection implemented as a null model (Comeron 2017).

### Differences in Efficiency of Selection across the *Leptidea* Species

The three species differed substantially in global estimates of *p*_N_/*p*_S_ and ω, with *L. sinapis* showing both a lower *p*_N_/*p*_S_ ratio and a lower ω than *L. reali* and *L. juvernica*, indicating more efficient selection against segregating slightly deleterious nonsynonymous polymorphisms in this species. We also observed a slightly higher proportion of adaptive nonsynonymous substitutions in *L. sinapis* than in *L. reali*. This is consistent with the overall higher genetic diversity in *L. sinapis* (θ_π_ = 0.0031) than in *L. reali* (θ_π_ = 0.0024) and *L. juvernica* (θ_π_ = 0.0016), suggesting a larger long-term *N*_e_ (Talla et al. 2017, 2019). However, all species had relatively high *p*_N_/*p*_S_ and ω compared with other insect taxa (Heger and Ponting 2007; Rouselle et al. 2016; Okamura et al. 2019; Pinharanda et al. 2019), indicating less efficient selection against slightly deleterious alleles in *Leptidea*. This is in line with the low overall genetic diversity in all investigated species, supporting comparatively low long-term *N*_e_.

### Associations between Base Composition, Genetic Diversity, and Recombination

We found a higher level of polymorphisms in 4D sites than in introns and intergenic sequences in *Leptidea*. This has also been observed in other species. For example, genetic diversity in 3rd codon positions was elevated compared with introns in humans and the Pacific oyster (*Crassostrea gigas*), suggesting that introns contain conserved regions important for gene regulation and splice site recognition (Amit et al. 2012; Song et al. 2018). In our case, this pattern was altered when only GC conservative mutations were analyzed (weak to weak (A/T) or strong to strong (G/C)), and 4D sites had a similar or lower level of genetic diversity than introns and intergenic sequences for these mutation categories. These observations suggest that genetic diversity in 4D sites is determined both by a reducing effect of linked selection from nearby nonsynonymous sites, and an inflating effect caused by a higher mutation frequency as a consequence of a higher GC-content in 4D sites than in any other site category. Spontaneous deaminations generally induce mutations at a higher rate from G/C to A/T than vice versa, meaning that GC rich regions should accumulate novel mutations at a higher rate than GC poor regions leading to an equilibrium GC-content <50% (Gojobori et al. 1982; Petrov and Hartl 1999; Lynch 2010; Duchêne et al. 2015).

We found only weak correlations between GC-content and ρ in all populations. This may be due to at least three reasons. First, we do not expect to see a strong correlation between GC-content and ρ if GC-biased gene conversion (gBGC) is absent in our investigated taxa, even if there is extensive regional variation in recombination frequency. gBGC is a neutral process that results from preferential transmission of G/C over A/T bases during double strand break repair in heteroduplex DNA with G/C to A/T mismatches (Marais 2003; Duret and Galtier 2009; Pessia et al. 2012). Second, since ρ was estimated in 100 kb windows, the presence of recombination hot-spots may have been overlooked, since such regions, at least in some organisms, can occur on a much narrower scale (McVean et al. 2004; Singhal et al. 2015). This could lead to a diluted association between recombination and base composition. However, the fact that we detected a significant effect of recombination rate on regional variation in genetic diversity indirectly indicates that the lack of a strong correlation between recombination and GC is not due to technical caveats in estimating ρ. Third, even if gBGC occurs in *Leptidea*, an even recombination landscape and/or swift turnover of potential recombination “hot-spots” would result in weak correlations between GC-content and ρ and weak correlations in regional ρ estimates across populations. The data we have at hand for *Leptidea* are unfortunately not sufficient to discriminate between these scenarios and high-resolution recombination data and detailed quantitative analyses of gBGC are sparse in Lepidoptera (but see Galtier et al. 2018; Mackintosh et al. 2019; Martin et al. 2019).

### Recombination Rate, Gene Density, and Variation in Diversity

The *Leptidea* species differ extensively in chromosome numbers and there is extreme intraspecific variation in karyotype setup (2*n* range from ∼56 to ∼108) in *L. sinapis* (Dincă et al. 2011; Lukhtanov et al. 2011; Šíchová et al. 2015). If correct chromosome segregation is dependent on crossing over (Storlazzi et al. 1995; Pardo-Manuel de Villena and Sapienza 2001; Wang et al. 2015), we would expect that populations with a larger number of chromosomes had elevated global recombination rates (Mackintosh et al. 2019). We found only minor differences in global ρ between species and populations (with the exception of LjIre), indicating that the total number of crossovers is similar across populations. In contrast to the expectations from an association between genome-wide recombination rate and genetic diversity, the overall levels of diversity were higher in LsSwe and LsKaz (2*n* ∼ 56–60) than in LsSpa (2*n* ∼ 106–108). Taken together, these observations suggest that the genome-wide recombination rate is not directly affected by chromosome fissions and fusions. A potential explanation for this is that correct segregation of chromosomes during meiosis can occur without chiasma formation. In Lepidoptera, there is female achiasmy (Turner and Sheppard 1975; Suomalainen et al. 2009; Suomalainen 2010)—that is, no recombination resulting in crossover of parental chromosomes occurs in females. It is not known if chiasma formation still occurs in females but that recombination is resolved without crossing over (noncrossover)—the predominant outcome of recombination in eukaryotes (Hillers 2004; Smeds et al. 2016). Alternatively, even if crossing-over is necessary for correct segregation in male meiosis and chiasma formation occurs in females in Lepidoptera, it is possible that karyotypic changes have limited impact on the overall recombination rate. For instance, if interference mechanisms which generally regulate the spatial distribution to reduce nearby crossover events (Hillers 2004) are absent or less rigorous, the overall recombination rate might not be affected unless fissions result in very small chromosomes where interference plays a significant role. The latter explanation would suggest that the global recombination rate could be high in Lepidoptera as compared with other taxa, but detailed direct estimates of recombination will be needed to verify this. As discussed earlier, ρ is a product of *r* and *N*_e_. LsSpa most likely has had lower long-term *N*_e_ than LsSwe and LsKaz (Talla et al. 2019), and we cannot rule out that differences in *N*_e_ across *L. sinapis* populations may mask a potential effect of karyotypic differences.

In the MLR analysis, we also noted that the relative effects of recombination rate (ρ), gene density, and mutation rate (*d*_S_) varied between populations. In populations with higher genetic diversity (LsSwe, LsKaz, LsSpa, and LjKaz), recombination rate was the main factor while the mutation rate was the most significant factor in LrSpa and LjIre, populations with lower genetic diversity in general (Talla et al. 2019). This is in line with a stronger effect of linked selection in populations with larger *N*_e_ and that diversity in smaller populations is more dependent on mutational input. However, the association might potentially trace back to the increased power to detect recombination events in populations with higher diversity.

### Annotation of the *Leptidea sinapis* Genome Assembly

The number of annotated genes in the *L. sinapis* genome (15,598) is similar to most available annotated moth and butterfly genomes: for example, *Bombyx mori* (15,488 genes), *Calycopis cecrops* (16,456), *Danaus plexippus* (15,130 genes), *Heliconius erato* (13,676), *Lerema accius* (17,411), *Papilio glaucus* (15,692), *Papilio machaon* (15,497), *Papilio xuthus* (15,322), and *Phoebis sennae* (16,117), with the exception of *Bicyclus anynana* (22,642), *Heliconius melpomene* (20,102), and *Papilio polytes* (12,244) (Challis et al. 2017). Hence, it is likely that the core gene set in Lepidoptera consists of ∼15,000 genes, slightly lower than the gene set in, for example, *Drosophila* (≈17,000) and humans (≈21,000). Based on orthology searches in databases, ∼2/3 of the genes had functional information from other taxa. It should be noted that most information was taken from distantly related taxa (predominantly from *D. melanogaster*) and direct inference of functions obviously have to be verified within *Leptidea* if genotype–phenotype interactions are to be established. Since most gene functions are conserved over deep time scales, an initial idea about relevance of specific gene classes—in our case, genes under positive selection in different *Leptidea* species and populations—can still be achieved.

## Conclusions

In this study, we used annotation and recombination data together with available whole-genome resequencing data from 60 individuals of three different species of butterflies to investigate the effects of mutation, recombination, and selection on regional levels of genetic diversity. We found that genome-wide diversity and rate of adaptive evolution was comparatively low, and the ratio of nonsynonymous to synonymous polymorphisms and substitutions comparatively high, in line with small long-term effective population sizes. Still, physical linkage, predominantly via the effect of background selection has resulted in reduced diversity in regions where gene density is high and/or the recombination rate is low in all species.

## Supplementary Material

Supplementary data are available at *Genome Biology and Evolution* online.

## Supplementary Material

evz212_Supplementary_Data

## Notes

**Data deposition:** This project has been deposited at the European Nucleotide Archive (ENA) under the accession PRJEB21838. In house developed scripts and pipelines are available at: https://github.com/venta380/Leptidea_selection_project.
