# Supplementary material for: Dissecting the Effects of Selection and Mutation on Genetic Diversity in Three Wood White (Leptidea) Butterfly Species
Source: Genome Biol Evol. 2019 Oct 3;11(10):2875–86. doi: 10.1093/gbe/evz212 (PMC6795238; doi:10.1093/gbe/evz212)
Supplement: evz212_Supplementary_Data [file evz212_supplementary_data.pdf]

Supplementary Information.

Supplementary Methods

The annotation of the *L. sinapis* genome was computed with the Maker package, version 3.00.0 (Holt & Yandell, 2011) on the previously available genome assembly (Talla *et al.*, 2017). Before the annotation process was launched, different analyses were done in order to check the genome quality and to ensure the proper operation of the annotation pipeline. First, all scaffolds shorter than 10 kb were removed from the assembly since annotation rarely yield informative data for sequences < 10 kb. This reduced the number of scaffolds from 7,090 (Talla *et al.*, 2017) to 7,009. The completeness of the genome assembly was then assessed with BUSCO version v1.1b1 (Simao *et al.*, 2015). BUSCO provides measures for quantitative assessment of completeness of genome assemblies, gene sets, and transcriptomes. Gene sets contained within BUSCO are selected from orthologous groups with genes present as single-copy orthologs in at least 90% of the species in a specific taxonomic group. It includes 2,675 genes for arthropods, 3,023 for vertebrates, 843 for metazoans, 1,438 for fungi and 429 for eukaryotes. The results showed that 66% of the genes were complete in the *L. sinapis* genome assembly, 24% were partly fragmented, 9% were missing and 2.1% were duplicated. In addition, we applied repeat masking with both RepeatMasker version 4.0.3 (Smit *et al.*, 2013-2015) and RepeatRunner (http://www.yandell-lab.org/software/repeatrunner.html) using a previously established repeat library (leptidea_rm1.0_hexPlusMelpErato.li) (Talla *et al.*, 2017). The repeat masking process identified 1,279,916 repeats covering approximately 47% (302.3 Mb) of the *L. sinapis* genome assembly. The level of contiguity (7,009 scaffolds >10 kb, a large fraction of complete or partially fragmented genes) and conservative repeat masking assured that the annotation process could be applied.

The Maker package was run twice; the first time with RNA-seq and protein evidence data, that were used to train the prediction algorithm for subsequent *ab-initio* prediction, and a second time with both RNA-seq and protein evidence data and the *ab-initio* gene profiles. The RNA-seq libraries were first *de-novo* assembled using Trinity and the assembly was processed with an in-house developed pipeline (available at: https://github.com/LLN273/RNAseq_Lsinapis) including Trimmomatic (Bolger *et al.*, 2014), TopHat (Trapnell *et al.*, 2009) and StringTie (Pertea *et al.*, 2015). Two external protein data sets were used; one consisting of protein sequences from Uniprot (https://www.uniprot.org/; accessed 2016-08-01) and the other was the *Heliconius melpomene* annotation (Heliconius_melpomene_melpomene_Hmel2_proteins.fa; 9,995 annotated coding sequences) available at LepBase (Challis *et al.*, 2017). The Uniprot database (Magrane & UniProt-Consortium, 2011) protein sequences were from the Swiss-prot section and contained only manually annotated and reviewed proteins (551,705 proteins).

As a basis for the construction of gene models, *ab-initio* predictions from four sources were used; Augustus (Stanke *et al.*, 2006), GeneMark_ES_ET (Ter-Hovhannisyan *et al.*, 2008), SNAP (Korf, 2004) and EVM (Haas *et al.*, 2008). The *ab-initio* annotation uses statistical models of genome composition to identify the most probable location of start/stop codons and splice sites. The *ab-initio* tools need to be trained in order to know how genes look like in the focal species (exon and intron lengths and numbers). Augustus version 2.7 (Stanke *et al.*, 2006) was trained with the annotation created after the first run of Maker. GeneMark-ES_ET version 4.3 (Ter-Hovhannisyan *et al.*, 2008), which has previously been shown to be efficient for unsupervised training on fungal and eukaryotic genomes (Lomsadze, 2005; Ter-Hovhannisyan *et al.*, 2008), was trained with the genome assembly as input. SNAP (Korf, 2004) was trained with the genome sequence and a selected set of high-quality genes from the evidence based

annotation. Maker (Holt & Yandell, 2011) was then run with all four predictors and combined with evidence based annotation. This strategy, using both evidence sequences (proteins and transcripts) and *ab-initio* predictions, allowed us to compute high-confidence gene models (Holt & Yandell, 2011).

Maker integrates an annotation quality-control called Annotation Edit Distance (AED) developed by the Sequence Ontology Project (http://www.sequenceontology.org/). The AED metric is a means to quantify the congruency between a gene annotation and its supporting evidence. We omitted all gene predictions with AED = 1 since this indicates a pure *ab-initio* gene model without evidence from previously available RNA-seq or protein sequence data. Statistical evaluation of the final annotation was performed with an in-house developed perl script (available at: https://github.com/NBISweden/GAAS). The annotation identified 15,598 protein coding genes with an average coding sequence length (CDS) of 1,134 bp (Supplementary Table 1). BUSCO version v1.1b1 (Simao *et al.*, 2015) was run again after the structural annotation with the set of proteins predicted by the annotation process. The result showed that around 69% (n = 1,860) of the genes in the arthropod gene set (n = 2,675) were complete in the genome, 14% (400) were fragmented, 15% (415) were missing and 11% (319) were duplicated. Part of the number of duplicated copies can be explained by isoforms that are sometimes counted as duplicated genes.

Functional inference for genes and transcripts was performed using the translated CDS features of each coding transcript. Each predicted protein sequence was blasted against the Uniprot/Swissprot reference data set (downloaded 2016-08) in order to retrieve the gene name and the protein function. The predicted gene sequences were also run with InterProscan version 5.7-48 (Jones *et al.*, 2014) in order to retrieve Interpro (Hunter *et al.*, 2012), PFAM (Finn *et*

al., 2014), GO (Ashburner *et al.*, 2000), MetaCyc (Caspi *et al.*, 2018), KEGG (Kanehisa *et al.*, 2014) and Reactome (Fabregat *et al.*, 2018) data. Finally, we used outputs from those analyses using the Annie annotation tool (Tate *et al.*, 2014) to extract and reconcile meta data into predictions for canonical protein names and functional predictions. The combined analyses of protein domains resulted in 10,857 genes with and 4,741 genes without functional annotation information (Supplementary Table 2). The algorithm implemented in tRNAscan version 1.3.1 (Lowe & Eddy, 1997) was used to predict tRNA genes in the *L. sinapis* genome assembly. We found a large set of tRNAs (n = 22,961) but only a fraction of these (n = 51) were supported by both evidence and *ab-initio* models.

Supplementary Table 1.

The number of SNPs before and after quality filtering (GQ < 15 and MQ < 20) for each of the six populations analysed.

Population	Total # SNPs	# SNPs after filtering
**LsSwe**	6,087,199	6,086,844
**LsKaz**	5,308,021	5,307,513
**LsSpa**	5,810,369	5,808,814
**LrSpa**	4,209,882	4,207,729
**LjKaz**	3,818,134	3,798,864
**LjIre**	2,262,560	2,258,959

Supplementary Table 2.

Summary of number of protein coding genes (# genes) found in the *Leptidea sinapis* genome annotation. Average length of the coding region across all genes (CDS length), proportion of genes with predicted 5' and 3' UTR regions (UTR, in %) and the fraction of the entire assembly length covered by protein coding genes including UTRs (Genomic coverage, in %).

# genes	CDS length	UTR	Genomic coverage
15,598	1,134	72.11%	3.7%

Supplementary Table 3.

Statistics about functional and protein domain annotations retrieved in a selection of different databases. PFAM (Protein families) is a large collection of multiple sequence alignments for many common protein domains and families across different organism groups. Interpro integrates diverse information about protein families, domains and functional sites. Central to the database are diagnostic models, known as signatures, against which protein sequences can be searched to determine their potential function. The GO (Gene Ontology) project has developed three structured, controlled vocabularies (ontologies) that describe gene products in terms of their associated biological processes, cellular components and molecular functions in a species-independent manner. KEGG, MetaCy and Reactome are databases providing gene pathway information. Total = total number of *L. sinapis* genes with functional information from one or several databases. NF = number of *L. sinapis* genes that did not match any functionally annotated gene in any database.

	**PFAM**	**Interpro**	**GO**	**KEGG**	**MetaCy**	**Reactome**	**Total**	**NF**
**# genes**	9,688	10,805	8,113	724	477	2,632	10,857	4,741

Supplementary Table 4.

The observed GC content in different site categories in the *L. sinapis* genome assembly. CDS = protein coding (exonic) sites. The genome-wide estimate includes all sites (protein coding + non-coding) in the *L. sinapis* assembly.

**Category**	**GC content (%)**
CDS	44.9 ± 8.4
Genome-wide	31.6 ± 2.7
1st codon position	47.3 ± 7.1
2nd codon position	43.1 ± 6.9
3rd codon position	45.8 ± 9.1
4D-sites	58.9 ± 13.1
Introns	30.7 ± 6.5
Intergenic sequence	31.4 ± 4.7

Supplementary Table 5.

Summary of gene density estimates (percentage of protein coding / exonic sites) in low- (Low) and high (High) diversity regions in all species and significance levels (P-value) for each respective Mann-Whitney *U*-test.

**Species**	**Low**	**High**	**P-value**
L. sinapis	5.63 ± 3.63	5.28 ± 3.05	$3.7*10^{-09}$
L. reali	5.65 ± 3.49	5.24 ± 3.11	$2.7*10^{-09}$
L. juvernica	5.77 ± 3.32	5.63 ± 3.60	$2.1*10^{-03}$

Supplementary Table 6.

Summary of GC content (% guanine and cytosine nucleotides) in low- (Low) and high (High) diversity regions in all species and significance levels (P-value) for each respective Mann-Whitney *U*-test.

**Species**	**Low**	**High**	**P-value**
L. sinapis	31.3 ± 2.9	31.9 ± 2.5	$1.2*10^{-14}$
L. reali	31.4 ± 2.9	31.9 ± 2.5	$1.2*10^{-11}$
L. juvernica	31.3 ± 2.9	31.9 ± 2.5	$2.5*10^{-12}$

Supplementary Table 7.

A) Summary of the multiple linear regression analysis where base composition (GC), recombination rate (ρ), gene density (GD) and mutation rate (d_S) were used as explanatory variables for variation in genetic diversity at 4-fold degenerate coding positions (π_{4D}). B) The variance inflation factors for each of the explanatory variables (rows) used in the multi-linear regression (MLR) analysis (Table 2)for each respective population (columns).

A

**LsSwe**	Estimate	Std. Error	t value	Pr(>|t|)	
(Intercept)	$6.48*10^{-2}$	$5.09*10^{-4}$	127.16	$< 2.0*10^{-16}$	***
GC	$-7.94*10^{-5}$	$4.94*10^{-4}$	-0.16	0.872	
ρ	$2.12*10^{-3}$	$5.06*10^{-4}$	4.18	$2.98*10^{-5}$	***
GD	$-7.40*10^{-4}$	$5.32*10^{-4}$	-1.39	0.165	
d_S	$-2.41*10^{-2}$	$2.72*10^{-2}$	-0.89	0.374	
GC : ρ	$-6.56*10^{-4}$	$4.34*10^{-2}$	-1.51	0.131	
GC : GD	$-2.68*10^{-4}$	$4.86*10^{-4}$	-0.55	0.582	
ρ : GD	$2.42*10^{-4}$	$5.09*10^{-4}$	0.48	0.635	
GC : d_S	$-5.03*10^{-2}$	$2.92*10^{-2}$	-1.73	0.085	
ρ : d_S	$-2.01*10^{-2}$	$2.82*10^{-2}$	-0.71	0.476	
GD : d_S	$-3.28*10^{-2}$	$2.77*10^{-2}$	-1.18	0.236	
GC : ρ : GD	$-9.16*10^{-5}$	$3.30*10^{-4}$	-0.28	0.781	
GC : ρ : d_S	$-6.39*10^{-3}$	$2.70*10^{-2}$	-0.24	0.813	
GC : GD : d_S	$3.57*10^{-2}$	$3.00*10^{-2}$	1.19	0.234	
ρ : GD : d_S	$-1.93*10^{-2}$	$2.72*10^{-2}$	-0.71	0.479	

**LsKaz**	Estimate	Std. Error	t value	Pr(>|t|)	
(Intercept)	$6.22*10^{-2}$	$5.11*10^{-4}$	121.67	$< 2.0*10^{-16}$	***
GC	$1.51*10^{-4}$	$4.98*10^{-4}$	0.30	0.762	

ρ	$9.59*10^{-4}$	$5.09*10^{-4}$	1.88	0.060	
GD	$-7.45*10^{-4}$	$5.35*10^{-4}$	-1.39	0.164	
d_S	$-2.29*10^{-2}$	$2.71*10^{-2}$	-0.84	0.399	
GC : ρ	$4.81*10^{-5}$	$4.75*10^{-4}$	0.10	0.919	
GC : GD	$-5.33*10^{-7}$	$4.95*10^{-4}$	0.00	0.999	
ρ : GD	$-1.00*10^{-4}$	$5.18*10^{-4}$	-0.19	0.846	
GC : d_S	$-6.82*10^{-2}$	$2.85*10^{-2}$	-2.40	$1.70*10^{-2}$	*
ρ : d_S	$1.50*10^{-2}$	$2.66*10^{-2}$	0.56	0.572	
GD : d_S	$-4.01*10^{-2}$	$2.79*10^{-2}$	-1.44	0.151	
GC : ρ : GD	$4.20*10^{-2}$	$3.59*10^{-4}$	1.17	0.242	
GC : ρ : d_S	$-1.00*10^{-2}$	$2.91*10^{-2}$	-0.34	0.731	
GC : GD : d_S	$3.76*10^{-2}$	$3.08*10^{-2}$	1.22	0.223	
ρ : GD : d_S	$6.10*10^{-2}$	$2.66*10^{-2}$	2.29	$2.20*10^{-2}$	*
**LsSpa**	Estimate	Std. Error	t value	Pr(>|t|)	
(Intercept)	$6.68*10^{-2}$	$4.98*10^{-4}$	133.96	$< 2.0*10^{-16}$	***
GC	$3.52*10^{-2}$	$4.86*10^{-4}$	0.73	0.469	
ρ	$5.22*10^{-3}$	$5.01*10^{-4}$	10.42	$< 2.0*10^{-16}$	***
GD	$2.78*10^{-4}$	$5.22*10^{-4}$	0.53	0.594	
d_S	$-6.19*10^{-2}$	$2.68*10^{-2}$	-2.32	$2.10*10^{-2}$	*
GC : ρ	$1.60*10^{-5}$	$4.66*10^{-4}$	0.03	0.973	
GC : GD	$-2.06*10^{-4}$	$4.77*10^{-4}$	-0.43	0.667	
ρ : GD	$-2.37*10^{-4}$	$4.85*10^{-4}$	-0.49	0.625	
GC : d_S	$-4.10*10^{-2}$	$2.77*10^{-2}$	-1.48	0.139	
ρ : d_S	$2.52*10^{-3}$	$2.60*10^{-2}$	0.10	0.923	
GD : d_S	$-1.78*10^{-2}$	$2.87*10^{-2}$	-0.62	0.535	
GC : ρ : GD	$-2.91*10^{-4}$	$3.84*10^{-4}$	-0.76	0.448	
GC : ρ : d_S	$2.18*10^{-2}$	$2.82*10^{-2}$	0.77	0.440	

GC : GD : d_S	$3.55*10^{-2}$	$3.07*10^{-2}$	1.16	0.247	
ρ : GD : d_S	$-8.89*10^{-3}$	$2.74*10^{-2}$	-0.33	0.745	
**LrSpa**	Estimate	Std. Error	t value	Pr(>|t|)	
(Intercept)	$6.37*10^{-2}$	$6.89*10^{-4}$	92.38	$< 2.0*10^{-16}$	***
GC	$-1.05*10^{-4}$	$6.67*10^{-4}$	-0.16	0.875	
ρ	$1.53*10^{-3}$	$6.62*10^{-4}$	2.31	$2.09*10^{-2}$	*
GD	$-1.28*10^{-3}$	$6.97*10^{-4}$	-1.84	$6.62*10^{-2}$	
d_S	-0.11	$1.85*10^{-2}$	-5.81	$7.17*10^{-9}$	***
GC : ρ	$-4.28*10^{-4}$	$5.86*10^{-4}$	-0.73	0.465	
GC : GD	$-1.25*10^{-4}$	$6.19*10^{-4}$	-0.20	0.840	
ρ : GD	$-9.35*10^{-5}$	$6.35*10^{-4}$	-0.15	0.883	
GC : d_S	$3.99*10^{-2}$	$1.83*10^{-2}$	2.18	$2.97*10^{-2}$	*
ρ : d_S	$1.89*10^{-2}$	$1.71*10^{-2}$	1.10	0.270	
GD : d_S	$-4.96*10^{-3}$	$1.87*10^{-2}$	-0.27	0.791	
GC : ρ : GD	$3.70*10^{-4}$	$3.72*10^{-4}$	0.99	0.321	
GC : ρ : d_S	$6.48*10^{-3}$	$1.69*10^{-2}$	0.38	0.702	
GC : GD : d_S	$1.83*10^{-3}$	$1.80*10^{-2}$	0.10	0.919	
ρ : GD : d_S	$2.43*10^{-2}$	$1.66*10^{-2}$	1.47	0.142	
**LjKaz**	Estimate	Std. Error	t value	Pr(>|t|)	
(Intercept)	$6.00*10^{-2}$	$5.44*10^{-4}$	110.33	$< 2.0*10^{-16}$	***
GC	$-7.35*10^{-4}$	$5.34*10^{-4}$	-1.38	0.169	
ρ	$4.30*10^{-3}$	$5.64*10^{-4}$	7.63	$3.52*10^{-14}$	***
GD	$-1.32*10^{-3}$	$5.74*10^{-4}$	-2.30	$2.15*10^{-2}$	*
d_S	$-8.69*10^{-2}$	$2.12*10^{-2}$	-4.09	$4.42*10^{-5}$	***
GC : ρ	$-5.07*10^{-4}$	$4.72*10^{-4}$	-1.07	0.283	
GC : GD	$6.65*10^{-4}$	$5.21*10^{-4}$	1.28	0.202	

ρ : GD	$5.54*10^{-4}$	$5.69*10^{-4}$	0.97	0.330	
GC : d_S	$4.28*10^{-2}$	$2.14*10^{-2}$	2.00	$4.58*10^{-2}$	*
ρ : d_S	$-1.33*10^{-2}$	$1.99*10^{-2}$	-0.67	0.504	
GD : d_S	$3.90*10^{-3}$	$2.06*10^{-2}$	0.19	0.850	
GC : ρ : GD	$-1.41*10^{-4}$	$3.51*10^{-4}$	-0.40	0.689	
GC : ρ : d_S	$4.97*10^{-2}$	$1.74*10^{-2}$	2.85	$4.41*10^{-3}$	**
GC : GD : d_S	$9.97*10^{-3}$	$1.87*10^{-2}$	0.53	0.595	
ρ : GD : d_S	$1.91*10^{-3}$	$1.84*10^{-2}$	0.10	0.917	
**LjIre**	Estimate	Std. Error	t value	Pr(>|t|)	
(Intercept)	$5.50*10^{-2}$	$5.42*10^{-4}$	101.53	$< 2.0*10^{-16}$	***
GC	$-4.64*10^{-4}$	$5.35*10^{-4}$	-0.87	0.385	
ρ	$6.90*10^{-4}$	$5.70*10^{-4}$	1.21	0.226	
GD	$-1.14*10^{-3}$	$5.69*10^{-4}$	-2.00	$4.5*10^{-2}$	*
d_S	$-6.06*10^{-2}$	$2.11*10^{-2}$	-2.88	$4.0*10^{-3}$	**
GC : ρ	$-2.92*10^{-4}$	$4.38*10^{-4}$	-0.67	0.505	
GC : GD	$-1.14*10^{-4}$	$5.12*10^{-4}$	-0.22	0.824	
ρ : GD	$-7.30*10^{-5}$	$6.07*10^{-4}$	-0.12	0.904	
GC : d_S	$-1.52*10^{-2}$	$2.07*10^{-2}$	-0.73	0.463	
ρ : d_S	$1.37*10^{-2}$	$2.24*10^{-2}$	0.61	0.540	
GD : d_S	$-2.08*10^{-2}$	$2.00*10^{-2}$	-1.04	0.298	
GC : ρ : GD	$-7.70*10^{-4}$	$3.83*10^{-4}$	-2.01	$4.5*10^{-2}$	*
GC : ρ : d_S	$4.15*10^{-2}$	$1.99*10^{-2}$	2.09	$3.7*10^{-2}$	*
GC : GD : d_S	$2.58*10^{-2}$	$1.80*10^{-2}$	1.43	0.153	
ρ : GD : d_S	$-1.39*10^{-2}$	$2.09*10^{-2}$	-0.67	0.505	

B

	LsSwe	LsKaz	LsSpa	LrSpa	LjKaz	LjIre
**Gene Density**	1.007	1.009	1.010	1.010	1.013	1.013
**GC-content**	1.008	1.010	1.006	1.010	1.005	1.023
**ρ**	1.008	1.011	1.008	1.005	1.010	1.019
d_S	1.002	1.001	1.003	1.005	1.012	1.010

Supplementary Figure 1.

Population recombination rate (ρ) estimates for each of the six populations. Distributions (and boxplots in top panel) show the density distributions of ρ calculated in 100 kb windows across the genome. LsSwe = brown, LsKaz = orange, LsSpa = red, LrSpa = blue, LjKaz = dark green and LjIre = light green.

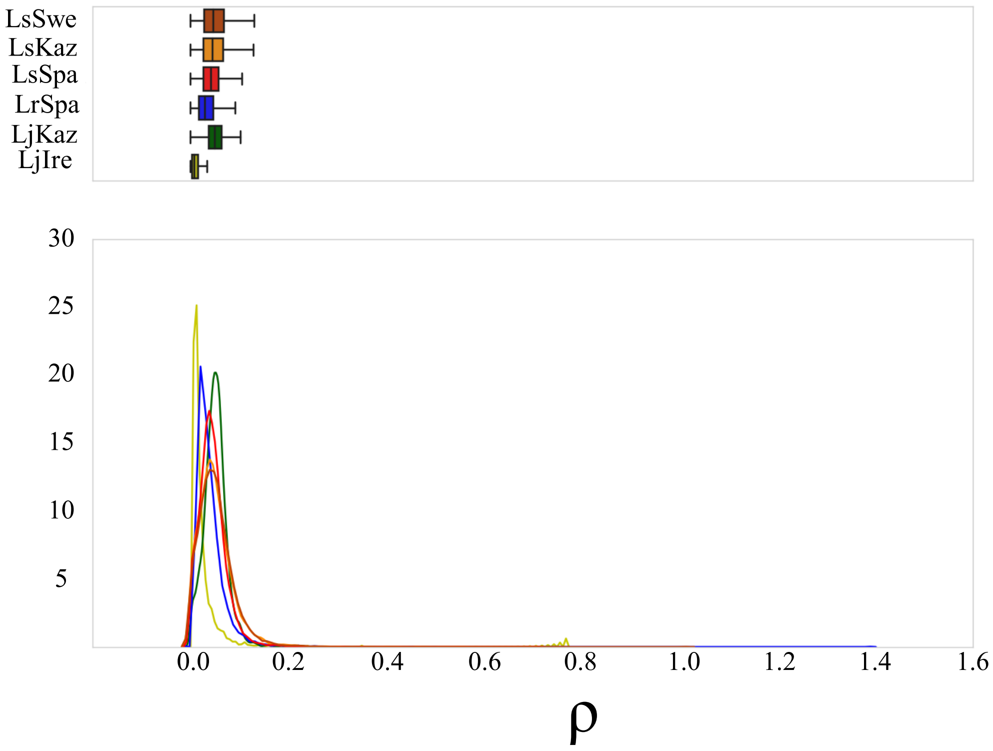

Supplementary Figure 2.

The associations between regional recombination rate (ρ) estimates for all 15 inter-population comparisons. The correlations were generally positive but weak (Pearson's r = -0.02 - 0.20, p-values > 0.05) with the exception of LsSwe vs. LsKaz where we found a significant positive correlation (Pearson's r = 0.21, p-value = $1.0*10^{-51}$).

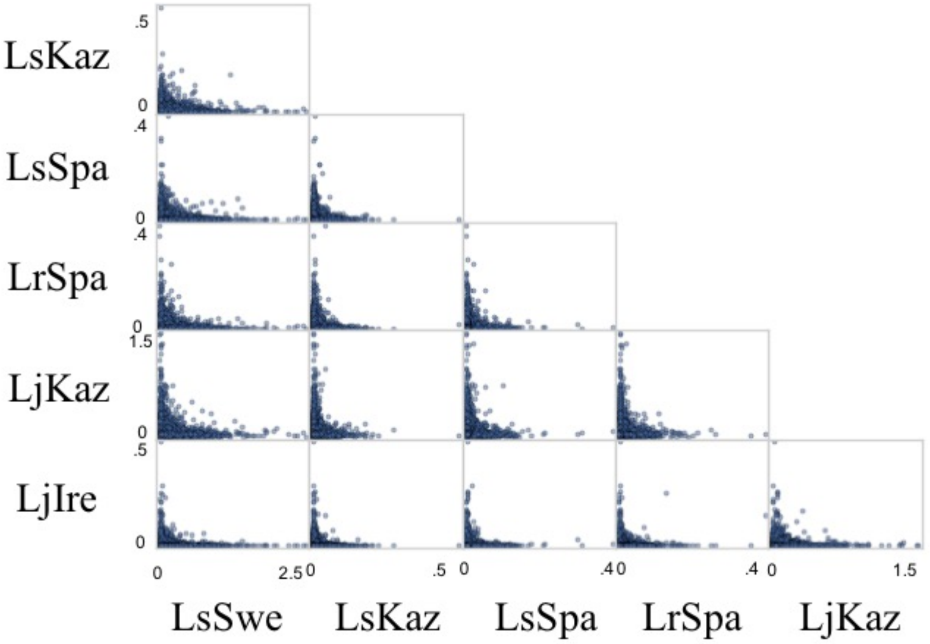

Supplementary Figure 3.

Population recombination rates and correlations with diversity. Populations in the following order (top left to bottom right): LsSwe (brown), LsKaz (red), LsSpa (orange), LrSpa (blue), LjKaz (dark green), LjIre (light green).

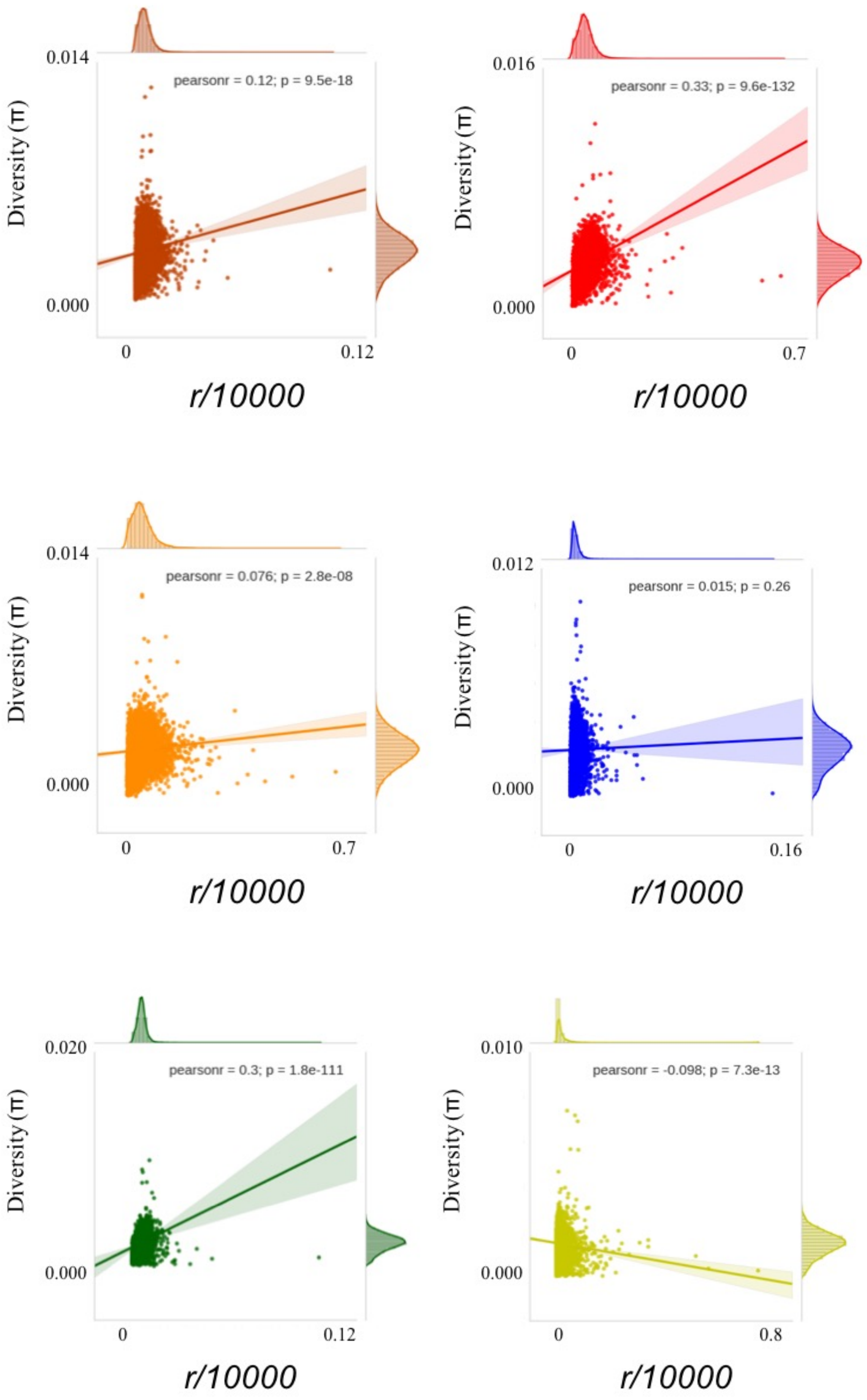

Supplementary Figure 4.

Ratio of non-synonymous to synonymous polymorphisms (p_N / p_S) calculated in 100 kb windows for low (relative diversity lower than 0, brown) and high diversity regions (relative diversity higher than 0, green). Note that this figure shows the same data as Table 1 in the main text but instead summarizes the data in box plots.

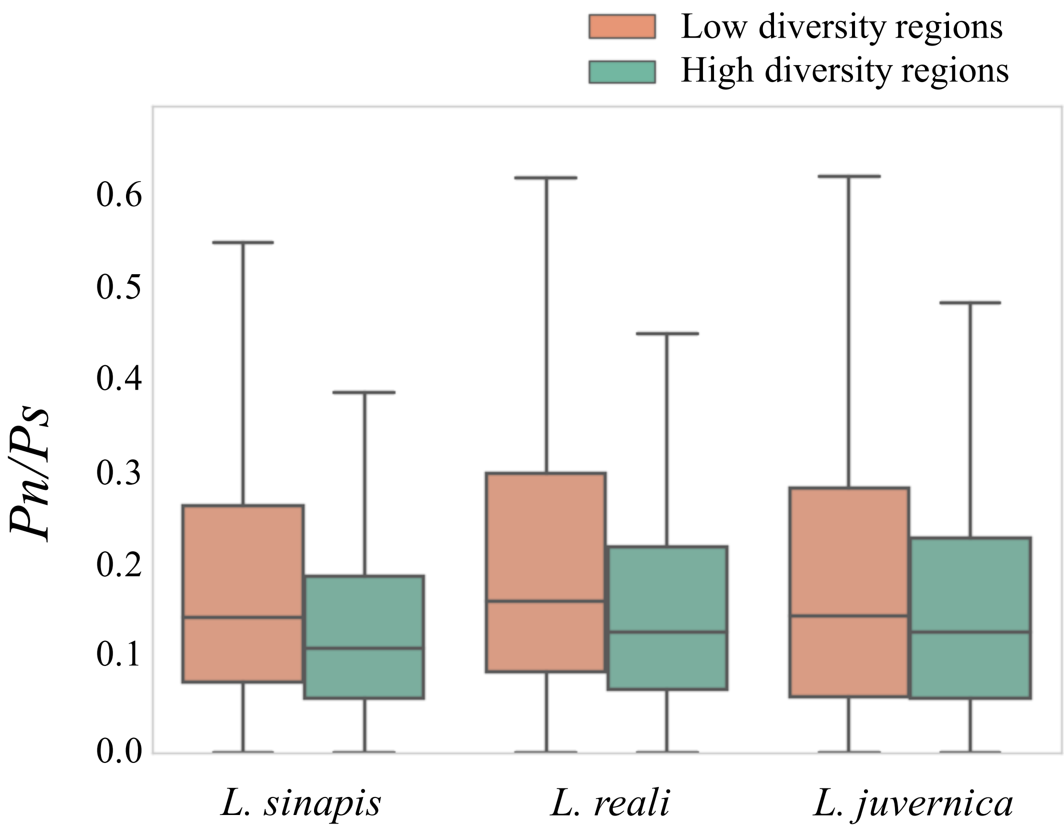

Supplementary Figure 5.

The ratio of non-synonymous to synonymous d_N / d_S (ω) plotted against the relative nucleotide diversity. The red line shows the mean ω of low diversity regions and the blue line shows the mean ω in the high diversity regions.

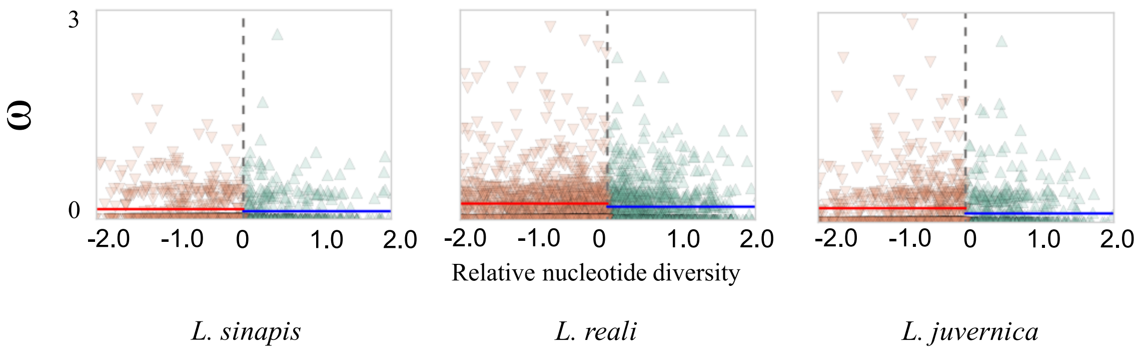

Supplementary Figure 6.

Boxplots showing the difference in gene density (proportion of protein coding / exonic sites in %) in low- (brown) and high (green) diversity regions. Exact numbers and significance levels are presented in Supplementary Table 5.

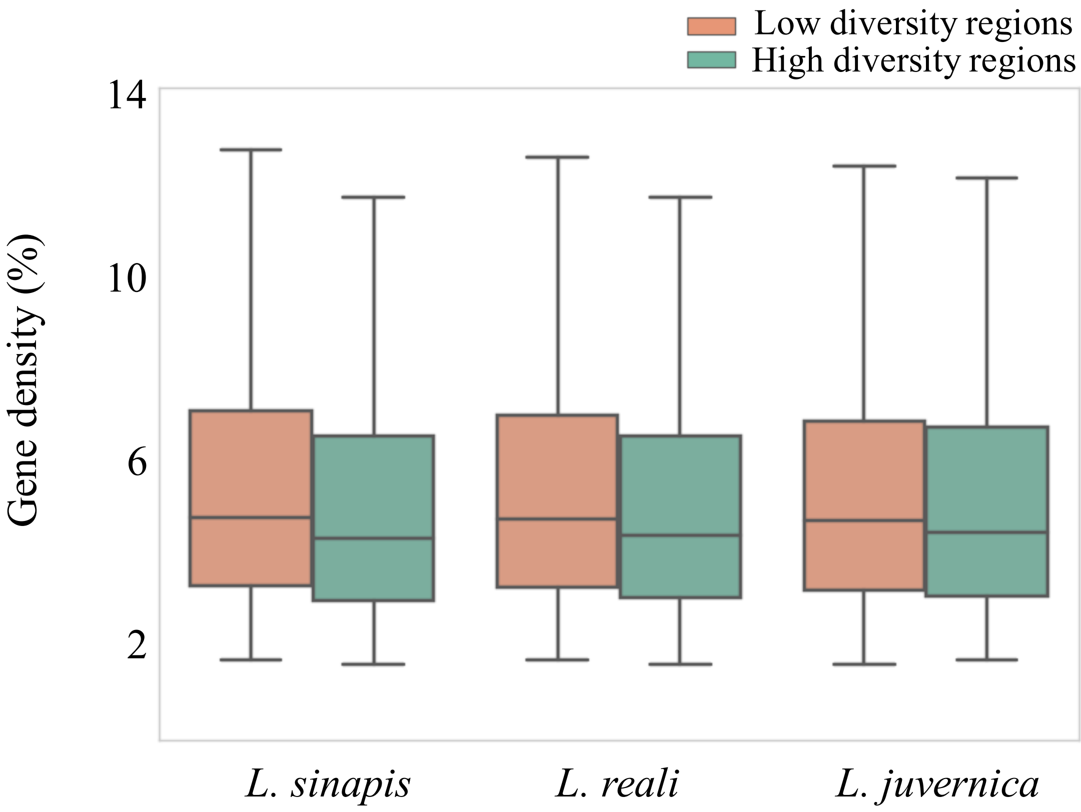

Supplementary Figure 7.

Boxplots showing the GC content (%) for low- (brown) and high (green) diversity regions. Exact numbers and significance levels are presented in Supplementary Table 6.

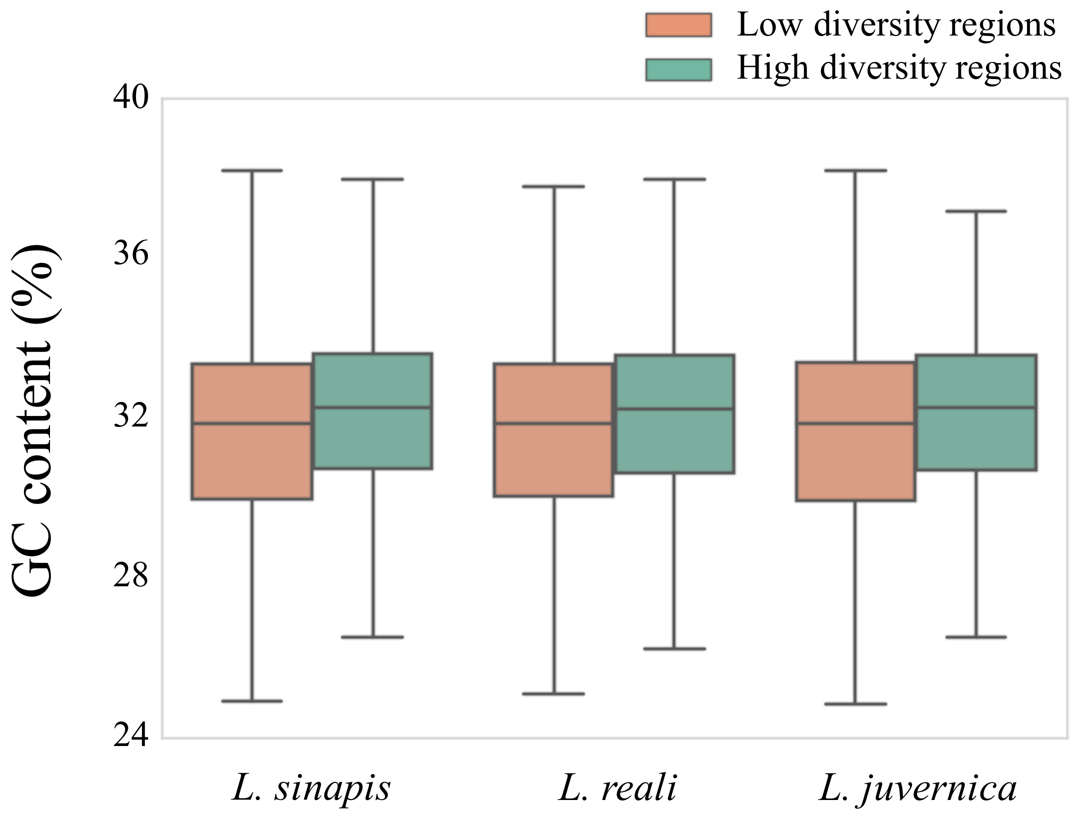

References

Ashburner, M., Ball, C. A., Blake, J. A., Botstein, D., Butler, H., Cherry, J. M., . . . Sherlock, G. (2000). Gene ontology: tool for the unification of biology. The Gene Ontology Consortium. *Nature Genetics, 25*, 25-29. doi:10.1038/75556

Bolger, A. M., Lohse, M., & Usadel, B. (2014). Trimmomatic: a flexible trimmer for Illumina sequence data. *Bioinformatics, 30*, 2114-2120. doi:10.1093/bioinformatics/btu170

Caspi, R., Billington, R., Fulcher, C. A., Keseler, I. M., Kothari, A., Krummenacker, M., . . . Karp, P. D. (2018). The MetaCyc database of metabolic pathways and enzymes. *Nucleic Acids Research, 46*, D633-D639. doi:10.1093/nar/gkx935

Challis, R. J., Kumar, S., Dasmahapatra, K. K., Jiggins, C. D., & Blaxter, M. (2017). Lepbase - the lepidopteran genome database. *BioRxiv, [Online version]*. doi:10.1101/056994

Fabregat, A., Jupe, S., Matthews, L., Sidiropoulos, K., Gillespie, M., Garapati, P., . . . D'Eustachio, P. (2018). The reactome pathway knowledgebase. *Nucleic Acids Research, 46*, D649-D655. doi:10.1093/nar/gkx1132

Finn, R. D., Bateman, A., Clements, J., Coggill, P., Eberhardt, R. Y., Eddy, S. R., . . . Punta, M. (2014). Pfam: the protein families database. *Nucleic Acids Research, 42*, D222-230. doi:10.1093/nar/gkt1223

Haas, B. J., Salzberg, S. L., Zhu, W., Pertea, M., Allen, J. E., Orvis, J., . . . Wortman, J. R. (2008). Automated eukaryotic gene structure annotation using evidencemodeler and the program to assemble spliced alignments. *Genome Biology, 9*, R7. doi:10.1186/gb-2008-9-1-r7

Holt, C., & Yandell, M. (2011). MAKER2: an annotation pipeline and genome-database management tool for second-generation genome projects. *BMC Bioinformatics, 12*, 491. doi:10.1186/1471-2105-12-491

Hunter, S., Jones, P., Mitchell, A., Apweiler, R., Attwood, T. K., Bateman, A., . . . Yong, S. Y. (2012). InterPro in 2011: new developments in the family and domain prediction database. *Nucleic Acids Research, 40*, D306-312. doi:10.1093/nar/gkr948

Jones, P., Binns, D., Chang, H. Y., Fraser, M., Li, W., McAnulla, C., . . . Hunter, S. (2014). InterProScan 5: genome-scale protein function classification. *Bioinformatics, 30*, 1236-1240. doi:10.1093/bioinformatics/btu031

Kanehisa, M., Goto, S., Sato, Y., Kawashima, M., Furumichi, M., & Tanabe, M. (2014). Data, information, knowledge and principle: back to metabolism in KEGG. *Nucleic Acids Research, 42*, D199-205. doi:10.1093/nar/gkt1076

Korf, I. (2004). Gene finding in novel genomes. *BMC Bioinformatics, 5*, 59. doi:10.1186/1471-2105-5-59

Lomsadze, A. (2005). Gene identification in novel eukaryotic genomes by self-training algorithm. *Nucleic Acids Research, 33*, 6494–6506. doi:10.1093/nar/gki937

Lowe, T. M., & Eddy, S. R. (1997). tRNAscan-SE: a program for improved detection of transfer RNA genes in genomic sequence. *Nucleic Acids Research, 25*, 955-964. doi: https://www.ncbi.nlm.nih.gov/pmc/articles/PMC146525/

Magrane, M., & UniProt-Consortium. (2011). UniProt knowledgebase: a hub of integrated protein data. In (Mar 29, 2011 ed.). Oxford Database.

Pertea, M., Pertea, G. M., Antonescu, C. M., Chang, T.-C., Mendell, J. T., & Salzberg, S. L. (2015). StringTie enables improved reconstruction of a transcriptome from RNA-seq reads. *Nature Biotechnology, 33*, 290-295. doi:10.1038/nbt.3122

Simao, F. A., Waterhouse, R. M., Ioannidis, P., Kriventseva, E. V., & Zdobnov, E. M. (2015). BUSCO: assessing genome assembly and annotation completeness with single-copy orthologs. *Bioinformatics, 31*, 3210-3212. doi:10.1093/bioinformatics/btv351

Smit, A., Hubley, R., & Green, P. (2013-2015). RepeatMasker Open-4.0. Retrieved from http://www.repeatmasker.org

Stanke, M., Keller, O., Gunduz, I., Hayes, A., Waack, S., & Morgenstern, B. (2006). AUGUSTUS: ab initio prediction of alternative transcripts. *Nucleic Acids Research, 34*, 435-439. doi:10.1093/nar/gkl200

Talla, V., Suh, A., Kalsoom, F., Dincă, V., Vila, R., Friberg, M., . . . Backström, N. (2017). Rapid increase in genome size as a consequence of transposable element hyperactivity in wood-white (*Leptidea*) butterflies. *Genome Biology and Evolution, 9*, 2491-2505. doi:10.1093/gbe/evx163.

Tate, R., Hall, B., DeRego, T., & Geib, S. (2014). Annie: the annotation information extractor. (Version 1.0) [Software].

Ter-Hovhannisyan, V., Lomsadze, A., Chernoff, Y. O., & Borodovsky, M. (2008). Gene prediction in novel fungal genomes using an ab initio algorithm with unsupervised training. *Genome Research, 18*, 1979-1990. doi:10.1101/gr.081612.108

Trapnell, C., Pachter, L., & Salzberg, S. L. (2009). TopHat: discovering splice junctions with RNA-seq. *Bioinformatics, 25*, 1105-1111. doi:10.1093/bioinformatics/btp120
